# Developing and Validating a Korean Version of the Assessment of Children’s Emotional Skills

**DOI:** 10.1007/s10578-022-01452-2

**Published:** 2022-10-13

**Authors:** C. Chung, S. Choi, J. Bae, H. Jeong, J. Lee, H. Lee

**Affiliations:** 1 School of Child Studies, Kyungpook National University, Daegu, South Korea; 2 Department of English Education, Kyungpook National University, Daegu, South Korea; 3 Department of Early Childhood Education, Keimyung College University, Daegu, South Korea; 4 Department of Home Economics Education, Kyungpook National University, Daegu, South Korea

**Keywords:** Social competence, Assessment of Children’s Emotional Skills, Validation, Emotional development

## Abstract

In this study, a Korean Assessment of Children’s Emotional Skills (ACES) was developed by modifying the original ACES which was initially introduced in the United States. Specifically, the original ACES was translated into Korean and revised to better fit the Korean cultural context. The content validity of the revised Korean ACES was established via expert reviews. To test its reliability, the revised Korean ACES was conducted on 286 six-year-old children. A confirmatory factor analysis indicated that our newly developed Korean ACES can be used as an appropriate tool to measure Korean children’s emotional skills. The Korean ACES can stimulate further studies on these emotional skills and contribute to various international collaborative studies that seek to compare the emotional skills of children from diverse cultural backgrounds.

## Introduction

Early childhood is a crucial time for emotional development and it forms the basis of social competence [1, 2]. It is the time during which emotional abilities can be acquired effectively as the recognition and understanding of emotions develop rapidly between ages three and six years [3–7]. Social competence refers to one’s ability to reach a goal by using one’s social skills and interacting with others [8]. It is widely considered an important part of early childhood social development and a predictor of children’s future ability to build social relationships [9–11]. A child’s development of social competence can help them successfully adjust to adult life in terms of mental health, wellbeing, and marriage in later life [12, 13]. Therefore, examining the relationship between social competence and emotional abilities in early childhood can help reveal individual differences in children's social competence and suggest directions for social and emotional education.

Among the emotional abilities, the skills to understand and recognize the emotions of others are reported to predict many future social outcomes [14–16]; thus, distinguishing emotional skills from other abilities is both theoretically and methodologically important [8]. The Assessment of Children’s Emotional Skills (ACES) was developed in the United States for this purpose in 2004, and our study modified this existing ACES tool to suit the Korean context. We then validated the Korean version of the ACES by administering the test to young Korean children.

### Children’s Emotional Skills and Social Competence

Young children experience a variety of social conflicts while interacting with their peers, and all children exhibit different levels of socially competent behavior in these situations [14]. Researchers have explored variables that might describe the processes by which children become socially competent. Studies about social competence have progressed along two parallel paths. One path has been followed by researchers who have focused on children’s emotional abilities, and the other by those who have focused on children’s social problem-solving skills and social information processing (SIP).

Many researchers have been interested in emotional abilities as the basis of social behavior [7, 17–21]. Emotional skill is a concept that includes emotion attribution and understanding. Children’s understanding of emotions (their emotional knowledge) is closely related to individual differences in social behaviors and adjustment [8, 14, 22–25]. Emotion researchers found that the ability to recognize and name emotions is related to social adaptation, peer relationships, and prosocial behavior [16, 23, 24, 26].

Several studies following the second path described earlier—the SIP model—have shown key explanatory power over the past 30 years. The basic premise of the SIP model is that children’s understanding and interpretation of social problem-solving situations influence their behavior [27]. The initial model (see Dodge, 1986, p. 84) assumes that cognitive biases or defects present at each step can lead children to develop or express maladaptive behaviors such as aggression, and emphasizes the importance of cognitive processing in social problem-solving [28, 29]. Subsequently, Crick and Dodge proposed a revised SIP model [30] (see p. 74) by supplementing the initial model. The model explains that children’s social behavior occurs through six stages: encoding of cues, interpretation of cues, clarification of goals, response access of construction, response decision, and behavioral enactment. While the initial model [28] emphasized only the importance of the cognitive process in social adaptation, it was revised through subsequent studies [29, 30] to include, emotional elements (e.g., emotional arousal, emotional state, and the experience of affect) as an important component of the SIP process. Even aggressive children may be less likely to interpret social cues as aggressive if they develop their emotional skills and learn to attribute the cause of emotional situations in different, less confrontational ways [25, 31].

In the same context, scholars have suggested that the development of social competence requires multilateral capabilities that integrate cognitive, emotional, and behavioral skills [32–34]. In general, emotions and cognition support the processing of information, and emotions are considered to have motivational, communicative, and accommodative functions within or between individuals in social competence, which may be distinguished from cognitive processes, such as attention, learning, deduction, and memory [27]. Recognition of emotion refers to recognizing another person’s emotional state through affective cues such as facial expressions, vocal cues (e.g., tone), or physical cues. Understanding emotions refers to understanding the situations in which certain emotions occur and how people’s internal states change in reaction to external circumstances and manifest as emotions and behaviors [35].

Lemerise and Arsenio [27] suggested the integrated SIP model (see p. 113) by adding emotional processes to the revised SIP model. Their model also includes various emotional factors, such as the affective nature of children’s relationships with their peers, their empathic responsiveness, and their production of emotions at each step of the revised SIP model. The integrated SIP model agrees with the revised SIP model that in the first two steps of SIP, children encode and interpret social situations according to both their internal emotions and external social cues (including affective cues from others). Thus, in both models, emotional signals constantly provide information on how social situations are developing and help children adjust their behavior.

The integrated SIP model suggests that specific factors—affective cues from peers, emotion attribution, and empathic responsiveness—are impactful in the first step of SIP, and thereby enhance the role of emotions in encoding social cues. In the integrated SIP model, it was argued that other people's emotional cues are also important sources of information and should be encoded and interpreted together [27]. Emotional cues from self and others provide constant information about how social situations are progressing, enabling them to sensitize their behavior; that is, how accurately one recognizes and understands the emotions of others in the stage of encoding of the clues affects not only the interpretation of these clues but also the subsequent SIP process. Aggressive children, for example, are more likely to think that their friend has acted intentionally or hostilely in situations where their intentions are unclear, and the other person is angry [36]. Moreover, hostile interpretations of social cues by young children are related to thinking that the perpetrator was angry [37]. It is important for children to accurately recognize other people’s emotional states for them to competently engage in social situations; thus, children’s emotion attribution accuracy can predict their social competence [36]. Therefore, examining emotion attribution accuracy and the anger attribution bias in the encoding of the cues, which is the first stage of the SIP model, can be a key factor in understanding the SIP process of young children.

### Emotional Skills Assessment Tools

Despite the usefulness of Lemerise and Arsenio’s integrated SIP model [27], empirical studies on the model are lacking. Dodge and Rabiner [38] noted several limitations while evaluating the integrated SIP model, one of which was the problem of measurement in moral development. They claimed that developing an empirical measurement tool that can be clearly distinguished for each stage is the main task for subsequent research.

Studies of the SIP model in the Korean context are mostly based on the revised SIP model and assessed children’s SIP using the hypothetical‐situation instrument developed by Crick [39] and Crick and Dodge [40] and translated by Kim [41]. This instrument assesses only three steps of the revised SIP model—interpretation (Step 2), clarification of goals (Step 3), and response decision (Step 5). Thus, this way of validating the integrated SIP model has its limitations. It does not allow one to assess whether young children accurately recognize and understand other people's emotions in relation to the encoding of cues, which is emphasized in the integrated SIP model. Thus far, tools used to measure children’s emotional skills also measured children’s social and emotional abilities, such as social competence, rather than independently measuring emotional skills.

Existing tools for assessing children’s emotional abilities often incorporated emotional abilities with social and emotional skills. Denham [42] distinguished emotional competency skills from relational/prosocial skills in the social-emotional competency framework, but the term social and emotional skills has not yet been clearly defined [43]. Therefore, it is necessary to distinguish between emotional skills and social abilities, and according to the SIP model, it is necessary to empirically examine whether emotional skills are related to social abilities.

Humphrey et al. [44] found that all the tools that only measure children's emotional abilities (except ACES) were developed before the 2000s and a lot of time has passed since then. In Korea, the emotional intelligence scale developed by Lee [45] has been mainly used to examine children's emotional abilities. These tools for measuring young children’s emotional abilities mostly rely on parents’ or teachers’ observations and assessments of children’s emotional behavior, and do not include tools with which young children’s emotional skills can be directly measured. Researchers have argued that this kind of direct measurement is key for an accurate assessment of young children’s emotional abilities [4, 46, 47].

### The ACES

To measure children's emotional skills, this study sought to revise the ACES developed by Schultz et al. [36] to make it more suitable for the Korean sociocultural context and to test the Korean ACES on young Korean children. The ACES conceptualizes children’s emotional abilities as emotional skills and presents children’s emotion attribution accuracy and anger attribution bias as sub-factors. Children’s emotional attribution accuracy indicates how accurately they recognize and encode emotional signals from others [36]. Children’s anger attribution bias indicates their tendency to recognize or encode an emotion such as anger [22, 37, 48]. Anger is widely considered a core human emotion; thus, anger attribution bias in early childhood may lead children to develop aggressive behavior, delinquency, anxiety, depression, intimidation, and low self-esteem [49–51]. The original ACES contains 40 items spread across three sections (facial expressions, social situations, and social behaviors). Children’s scores in each of the three sections were summed and used to assess their emotion attribution accuracy and anger attribution tendencies.

### Cultural Specificity in Emotion Recognition

The original ACES comprises three sections: facial expressions, social situations, and social behaviors. Despite the theoretical utility of the ACES, applying the original tool to the Korean context has some limitations. Although universality and cultural specificity in emotional recognition have long been debated, studies have provided evidence of an in-group advantage (i.e., cultural bias) in judging emotions [52]. In particular, it has been reported that adults and children have racial biases in judging facial expressions of emotions [53, 54]; thus, it would be difficult to use the visual aids included in the original tool in the Korean context. For instance, emotional expressions depicted on faces of American children included in the tool may not accurately represent or reflect the same emotions for children in different cultures.

An in-group advantage has been found not only for the judgement of facial expressions but also in the task of judging emotions associated with nonverbal behavior [55]. Therefore, explanations of social situations and behaviors that include emotional stimuli cannot be generalized across cultural contexts. Measurement tools for developmental research should be repeatedly verified [6], but the methods themselves include an inherent assumption of what the culture is [56]. Accordingly, we developed and validated a Korean version of the ACES and examined the emotional development of Korean preschoolers.

## Methods

### Developing a Korean ACES

**Fig. 1:**
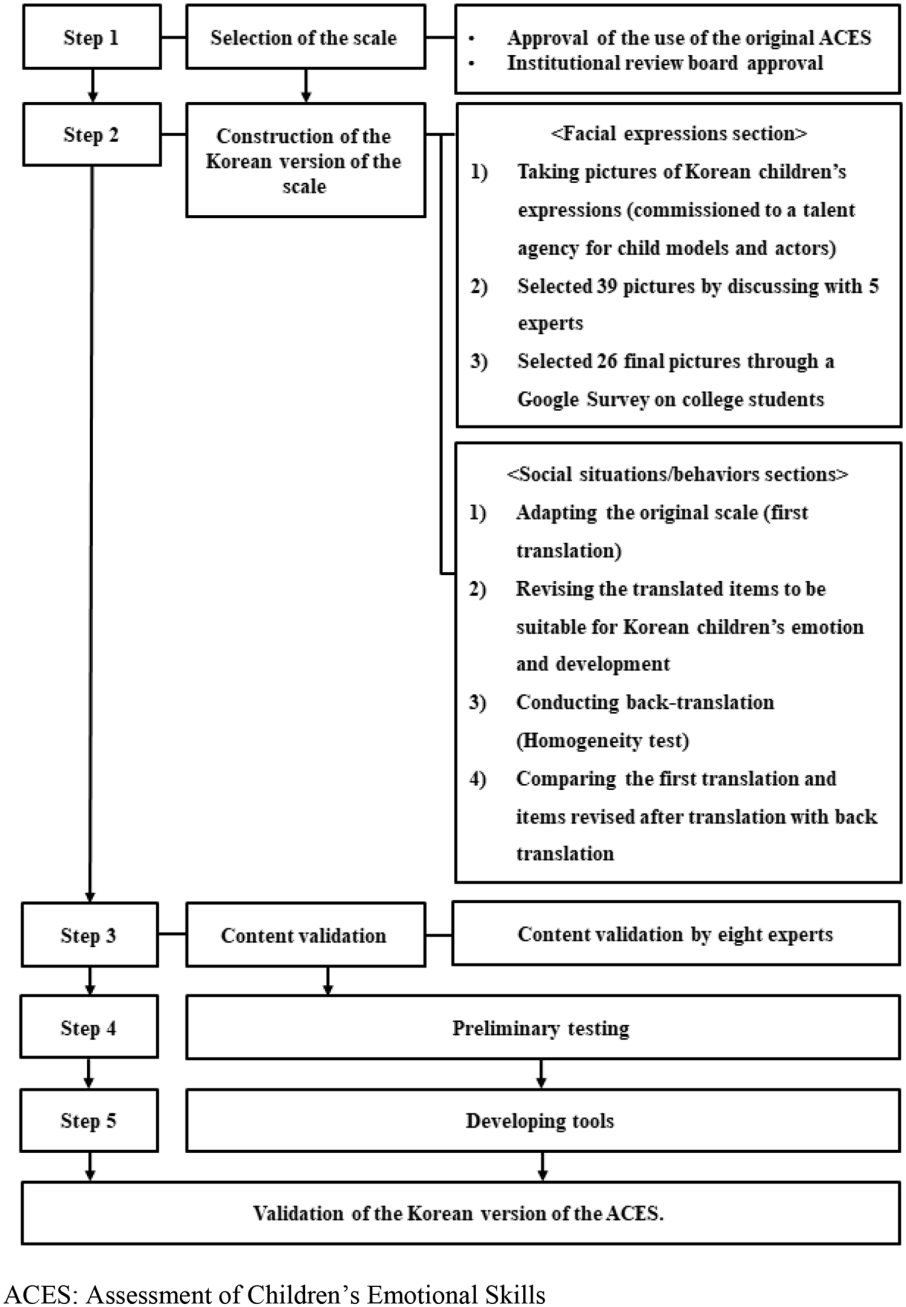
Procedure for developing and validating a Korean version of the ACES. ACES: Assessment of Children’s Emotional Skills

Our procedure for developing a Korean ACES is schematized in Fig. 1.

### Study Approval

Prior to this study, we explained its purpose and method to the original scale’s developer by e-mail. He sent us the pictures and script used to develop the original scale on Google Drive. This study was approved by the Institutional Review Board (no. 2019-0136) of Kyungpook National University.

### Constructing a Korean ACES

This section explains how we modified the items of the original ACES to create a Korean ACES.

#### Facial Expressions Section

The original ACES includes 26 images of preschool and elementary school children’s facial expressions. Of these 26 images, 16 show prototypical expressions of happiness, sadness, anger, and fear (four each), and the remaining 10 show mixed expressions of sadness and anger (to measure anger attribution). Given that previous studies have suggested that emotion recognition on the facial expressions are not universal [52, 57], we revised these images to Korean children’s facial expressions to validate our Korean ACES. To do this, we cast 20 child models from a talent agency for child actors and had them express emotions and expressions that were as similar as possible to the original scale’s pictures. We took a total of 3603 photos. We excluded shaky photos(361), photos showing the side of the face(2175), photos that hid the models’ eyebrows(522), and photos showing body parts other than the face (e.g., hands) (304). Five experts in early childhood education then discussed and selected six pictures each to represent children’s happiness, sadness, anger, and fear, and 15 pictures that represented a mix of sad ness and anger (to measure anger attribution) from the remaining 296 photos.

To verify typical facial expressions, the authors of the original scale asked 205 college students to evaluate photos of prototypical expressions of happiness, sadness, anger, and fear, and ambiguous mixes of some or each of these emotions. When making our final selection in this study, we conducted a Google Survey of 410 college students, who rated our 39 pictures of facial expressions for how prototypical they were of a given emotion. A total of 26 pictures were ultimately selected to be included in our final tool through this process.

#### Social Behaviors and Social Situations Sections

The social behaviors and social situations sections of the original ACES comprise 15 one-to-three-sentence-long statements. The social behavior items describe situations that express a prototypical emotion (e.g., someone skipping down the hall, whistling). There are three items each to represent happiness, sadness, anger, and fear, and three items that show a mix of emotions, to assess children’s anger attribution biases. The social situation items describe situations that induce prototypical emotions (e.g., someone is riding a bike, and as they ride downhill their speed increases beyond their comfort level). There are three items each describing the situations that might induce prototypical emotions of happiness, sadness, anger, and fear, and three items describing social situations that cannot be attributed to a sole individual emotion, to assess children’s anger attribution biases.

We completed the social behaviors and situations sections of the Korean ACES by translating and retranslating the items of the original scale. First, we had the original scale translated by a team of English education professors. The translated items were then partially revised by three professors of early childhood education (to ensure their suitability for Korean children). This Korean version was then translated into English by one foreign professor of English education who was bilingual and knew Korean cultural norms well because she had lived in Korea for a long time. Through this process, the items of the original scale were compared with the back-translated items, and those found to be inconsistent were translated and back-translated again to complete the second translation. Finally, we reviewed all items to ensure that they were both relevant and easily understood in Korean.

### Content Validation

To assess the content validity of the Korean ACES, we had eight experts (including three with PhDs in early childhood education, three PhD candidates in early childhood education, and two professors of early childhood education) review its contents. We explained our project and the tool to the experts by phone, secured their voluntary participation, and distributed the materials by e-mail. We had them rate whether the facial expressions were clearly distinguishable on a 5-point Likert scale (four pictures each) and whether the stimulus photos had an appropriate mix of sadness and anger (10 pictures). Their evaluations of the photos presented in the facial expression section indicated that emotions were well distinguished from facial expressions and ambiguous stimuli were also appropriately selected (*M* = 4.71, *SD* = 0.45). We also provided them with the original text and translation of the ACES and asked them to rate the adequacy of the translation and its suitability for young children on a 5-point Likert scale. For the social situations section, the translation was considered adequate (*M* = 4.75, *SD* = 0.57) and suitable for young children (*M* = 4.89, *SD* = 0.31). This was also true for the social behaviors section (adequacy of the translation: *M* = 4.54, *SD* = 0.56; suitability for young children: *M* = 4.54, *SD* = 0.62). Items with low scores were revised after consultation between researchers. Through this process, we found that all study materials were adequate for our purposes.

### Preliminary Testing

To examine whether our Korean ACES is suitable for Korean children, we conducted a preliminary test on four kindergarten children after obtaining their parents’ written consent to participate. We performed this test on October 16, 2019. The test was comprised of a one-on-one interview between a researcher and a child in a private indoor space. The child was presented with pictures on a laptop and their responses to the photos were marked on the questionnaire. The child was read the items of the social situations and behaviors sections and the child guessed the emotion of the person appearing in each item. These tests took about 30 min each; given that children would not be likely to concentrate through a long test, we split up the test into two sections: one for facial expressions and social situations and one for social behaviors. We eventually used printed photos to avoid eye fatigue from the laptop and provided emojis to help children express their responses.

### Developing Tools

**Fig. 2:**
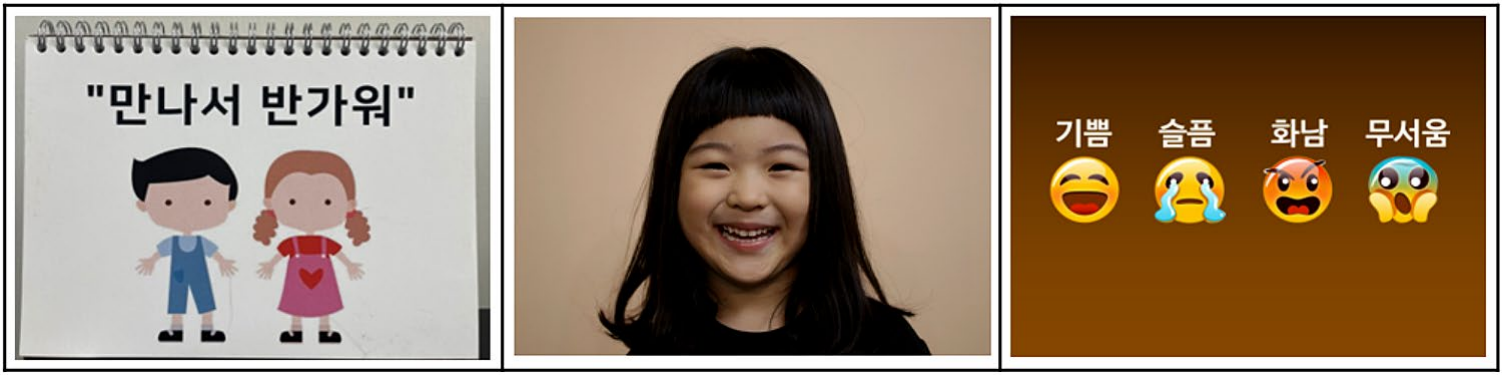
The calendar form for tools for the facial expressions section

Based on the results of the preliminary testing, we combined the pictures of facial expressions into the form of a calendar (Fig. 2). Our tool for assessing facial expressions was thus composed of two pictures of facial expressions for practice and 26 pictures for the facial expressions section of the Korean ACES. To help children express their emotions, we provided four emoticons (expressing happiness, sadness, anger, and fear), and presented these in random order so that children’s responses would not be affected by their order of presentation.

### Validating the Korean ACES

#### Participants

Participants were six-year-old children attending kindergartens and nurseries located in a metropolitan city in Korea. We selected institutions of early childhood education located in each district and county via convenience sampling. We then asked for their voluntary participation. Thirteen institutions consented to participate after we explained the purpose, content, and procedures of this study to the head of the institution and teachers. We distributed 538 copies of a consent form to secure children’s parents’ consent to participate. Of these, 322 were signed and returned to us. The study was conducted on all children who agreed to participate; however, 36 children who presented a language delay in the Preschool Language Receptive and Expressive Language Scale (PRES) test were excluded from the overall data. This left us with 286 participants for the final analysis—145 boys (50.7%) and 141 girls (49.3%). They were 76.12-months-old on average (SD = 3.51). In Korea, a new school year begins in March. Participants were kindergarteners.

#### Measurement

*Emotion skills.* The Korean ACES developed above is presented in Table 1. Children received one point when they provided accurate responses to facial expressions, situations, and behaviors, and zero points when they provided other emotions. Their overall scores were then divided by the number of items (40 items) to calculate their emotion attribution accuracy score. The tool also awards one point when children attribute an emotion to anger inappropriately (46 items) and zero points when they provide other emotions, to derive children’s anger attribution tendency score. Schultz et al., who devised the original ACES, found the reliability of the measurement tool (Cronbach’s α) to be 0.68 for emotion attribution accuracy and 0.66 for anger attribution bias [36].

**Table 1:** Construction of the ACES—number of items

Section	Facial expressions	Social situations	Social behaviors	Total
Emotion				
Happiness	4	3	3	10
Sadness	4	3	3	10
Anger	4	3	3	10
Fear	4	3	3	10
Ambiguous	10	3	3	16
No. of items	26	15	15	56

*Language abilities.* Given that previous studies indicated that children’s emotional skills are closely related to their language skills, three experts in language development conducted the PRES language test in a similar procedure. The PRES test was standardized by Kim et al. [58] on the Preschool Language Scale developed by Zimmerman and Steiner [59] to measure infant language ability. This tool was designed to evaluate the language development level of children aged 2–6 years. It consists of 45 items in receptive language and 45 items in expressive language (*N* = 90 items). Cronbach’s α within PRES subscales was 0.94 to 0.95, and the overall internal consistency was α = 0.95.

*Emotional intelligence.* To compare the Korean version of ACES with existing tools, the emotional intelligence measurement tool modified by Lee [45] was used. This tool was developed based on the emotional intelligence model proposed by Salovey and Mayer [21], and teachers evaluate children’s emotional intelligence. The 31 items addressing self-recognition and expression ability (*n* = 7, α = 0.81), self-regulation ability (*n* = 8, α = 0.94), ability to recognize others (*n* = 7, α = 0.91), and interpersonal ability to regulate others (*n* = 9, α = 0.87) are measured with a 6-point Likert scale. For the emotional intelligence measurement of children, 169 children (about 60% of the study participants) were evaluated.

*Social competence.* To measure the social competence of infants, a scale adapted by Chae [60] was used from LaFreniere and Dumas’ (1996) Social Competence and Behaviour Evaluation Inventory Short Form (SCBE-30) [61]. This 30-item tool is evaluated by teachers using a 6-point Likert scale: prosocial behavior (10 items, α = 0.86), depression–isolation behavior (10 items, α = 0.92), and anger–aggression behavior (10 items, α = 0.90).

#### Interviewer Training

Prior to the main test, we trained our interviewers to control for inspector effects. All interviewers had at least a master’s degree in early childhood education and experience working in a relevant institution. Training was conducted twice on October 18 and 21, 2019. During the training, interviewers received an explanation of the testing tool and were trained in relevant testing procedures. To ensure consistency, all inspectors were provided with a script about the procedures, such as greeting children, building rapport, explaining the test, conducting the test, and wrapping up.

#### Main Test

The main test was conducted between October 22 and December 18, 2019. Each child was interviewed one-on-one in a private space within their institution. The interviewers introduced themselves to the children and briefly explained what kind of test they would be taking. After this, children took the first test (measuring their reactions to facial expressions and social situations). The second test (social behavior and PRES) was conducted within two weeks, and all children who participated in the study were given a small toy worth USD $2 as reward. We asked the teacher of the children who completed the test with parental consent to rate the children’s social competence. Additionally, an emotional intelligence questionnaire was given to the teacher for about 70% of the participants.

### Data Analysis

We used SPSS 25.0 software to analyze the frequency of participants’ responses and calculate descriptive statistics for the sample. We also calculated reliability coefficients to determine the reliability of our Korean ACES. Next, we used AMOS 23.0 software to conduct a confirmatory factor analysis (CFA) to examine whether the Korean ACES effectively reflects the factor structure of the original scale. We used χ^2^/*df*, root mean squared error of approximation (RMSEA), the Tucker-Lewis index (TLI), and the comparative fit index (CFI) to determine model fit. We then analyzed descriptive statistics for emotion attribution accuracy, and anger attribution bias to investigate general trends in participants’ emotional skills, and to examine the correlation between the two subvariables. To examine the concurrent validity, the correlation coefficient with the emotional intelligence score evaluated by the teacher was calculated. To examine predictive validity, the correlation coefficient with the social competence score was calculated.

## Results

### Reliability Test

Cronbach’s αs were 0.64 for the 40 items representing prototypical emotions and 0.64 for the 46 items measuring anger attribution.

### CFA

**Fig. 3:**
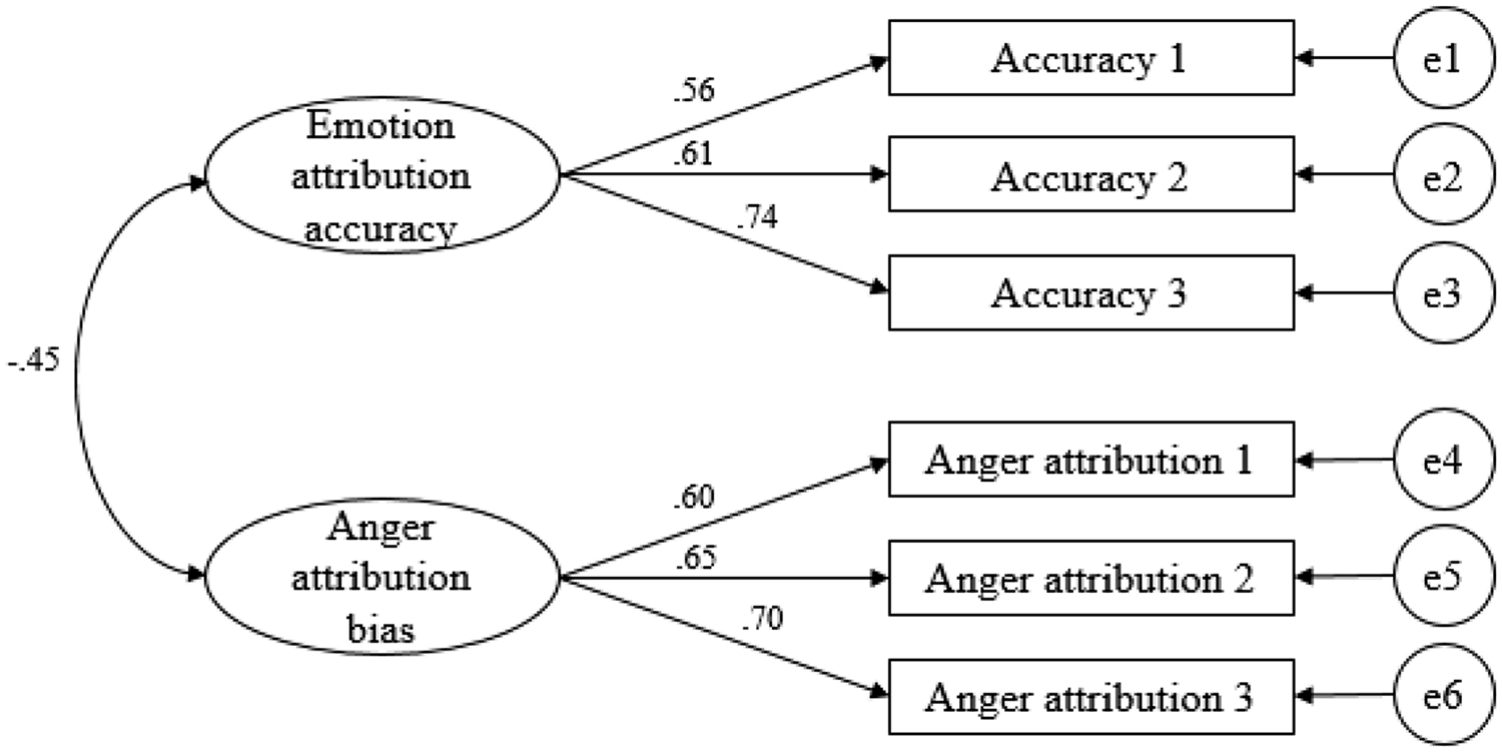
Models from the confirmatory factor analysis of ACES

We allocated the items of each section to each measurement variable for CFA and calculated the mean score of each item to create three observation variables for each subfactor. The results of the CFA are shown in Fig. 3. The model fit was favorable—χ^2^/*df* = 1.547, RMSEA = 0.044 (0.000 to 0.089), TLI = 0.096, CFI = 0.985. The standardized lambda value (estimate) of each measurement variable was at least 0.5, and the correlations among sub-factors were significant. That is, our Korean ACES reflects the factor structure of the original scale well, and its validity is supported.

### General Tendencies and Correlations Between Subvariables

We calculated descriptive statistics to examine the general tendencies of children’s emotional skills as specified by the Korean ACES (Table 2).

**Table 2:** Descriptive statistics of the ACES (*N* = 286)

	Min	Max	Mean	SD	Skewness	Kurtosis
Emotion attribution accuracy	0.40	0.95	0.76	0.09	− 0.46	0.00
Anger attribution bias	0.00	0.37	0.13	0.07	0.63	− 0.09

The mean of children’s emotion attribution accuracy was 0.76 (*SD* = 0.09) and the mean of their anger attribution bias was 0.13 (*SD* = 0.07). This indicates that our participants could recognize emotions with about 76% accuracy from social situations, behaviors, and facial expressions showing prototypical emotions, and that they recognized anger in about 13% of facial expressions, behaviors, and situations that do not clearly express anger. Pearson's correlation coefficient between the two subvariables was − 0.31; that is, the higher the emotional accuracy, the lower the anger attribution bias score.

### Concurrent Validity

To examine the concurrent validity of the Korean version of the ACES, correlation coefficients with emotional intelligence scores were calculated (Table 3). The accuracy of emotion attribution was positively correlated with self-recognition and expression ability (*r* = 0.20, *p* < 0.05) and interpersonal ability to regulate others (*r* = 0.16, *p* < 0.05). However, the relationship with anger attribution bias was non-significant.

**Table 3:** Correlations among the ACES, emotional intelligence and social competence

		ACES		Social competence		
		*A*	B	C	D	E
Emotional intelligence (*n* = 169)	F	0.20*	0.02	0.51**	− 0.54**	− 0.14
	G	0.09	0.01	0.57**	− 0.09	− 0.77**
	H	0.13	0.03	0.65**	− 0.40**	− 0.43**
	I	0.16*	0.04	0.76**	− 0.52**	− 0.52**
Social competence (*N* = 286)	C	0.13**	− 0.09			
	D	− 0.16*	0.05			
	E	− 0.12*	0.01			

A: Emotion attribution accuracy, B: Anger attribution bias, C: Prosocial behavior, D: Depression–isolation behavior, E: Anger–aggression behavior, F: Self-recognition and expression ability, G: Self-regulation ability, H: Ability to recognize others, I: Interpersonal ability to regulate others

*p < .05, **p < .01

### Predictive Validity

To examine the predictive validity of the Korean ACES, correlation coefficients with social competence scores were calculated (Table 3). The accuracy of emotion attribution was correlated with prosocial behavior (*r* = 0.13, *p* < 0.01), depression–isolation behavior (*r* = − 0.16, *p* < 0.05), and anger–aggression behavior (*r* = − 0.12, *p* < 0.05). However, the relationship with anger attribution bias was non-significant.

## Discussion

### Developing and Validating a Korean ACES

Our Korean ACES was adequately reliable and valid. This indicates that our tool can be used to measure Korean children’s emotional skills. However, this does not mean that it might be usefully translated and applied to other contexts; as discussed above, we accounted for a variety of sociocultural factors (facial expressions, language, and the like) when creating our version of the ACES, in accordance with studies that have shown that racial or cultural variables impact people’s ability to recognize emotion from facial expressions [52, 57, 62, 63]. Studies have emphasized that children’s emotional development is affected by the norms of the society they are raised in [64, 65]; thus, we cannot advise applying an unmodified version of our tool to another cultural context. Future studies should consider using our strategy of altering visual aids to fit a given cultural or racial context while still keeping the expressions and tool as similar to the original scale as possible.

We also verified that emotional recognition accuracy and anger attribution bias are components of children’s emotional skills. The results indicated that the factor structure presented by the authors of the original ACES was valid, and that there was a significant negative correlation between emotion attribution accuracy and anger attribution bias.

In the manuscript detailing the original ACES, which was developed in the US, emotional accuracy was reported to be 74% and anger attribution bias 14% [36]. For the Korean version, it was found that the averages of children's emotional attribution accuracy and anger attribution bias were similar to those among American children. Therefore, the Korean version of ACES appears suitably developed and validated in accordance with the cultural context of Korea while maintaining the structure of the original scale.

### Children’s Responses are More Accurate than the Teacher’s Evaluations

The ACES score showed a weak positive correlation with self-recognition and expression ability and interpersonal ability to regulate others in emotional intelligence. However, the correlation coefficient with self-regulation ability or other recognition ability was non-significant. In other recognition ability, items such as “the child knows well the mood of the person by looking at other people’s expressions” and “the child listens to the other person and knows the mood of the person well” are included. Comparing the accuracy of emotional recognition with this research tool that directly measured children’s emotional skills, it is a result that shows that the teacher’s measurement of children’s emotional skills may be inaccurate. In this study, the accuracy of emotional perception and anger attribution bias measured directly through ACES demonstrated a relatively small correlation with children's emotional intelligence and social ability measured by teachers, while the two variables measured by teachers showed a relatively high correlation. Since teachers have difficulty evaluating emotional ability, they might infer emotional ability by relying only on social behavior, thus explaining the high correlation between the two. That is, a teacher who observes a child with high anger-aggressive behavior may infer that the child has low emotional attribution ability, even if this is not actually the case. Compared to other tools, which primarily measure children’s emotional abilities through observations made by teachers or parents, our ACES tool attempts to measure children’s emotional skills directly. As mentioned in the literature review, researchers agree that the latter approach is better and much needed [46, 47]. However, most relevant measurement tools developed in Korea rely on teachers’ assessments of children’s behavior. Thus, we hope that our Korean ACES tests can further the cause of this kind of research in the Korean context.

### Emotion Attribution Accuracy, Anger Attribution Bias, and Social Competence

Like the claims that the emotional attribution accuracy and anger attribution bias can predict social adaptation [14, 22–24, 37], our results showed that emotional attribution accuracy was significantly correlated with prosocial behavior, depression–isolation behavior, and anger–aggression behavior. However, despite the claims that identifying children’s anger attribution biases can provide important cues for understanding (and preventing) children’s poor emotional and social development [36, 66, 67], anger attribution bias was not significantly correlated with children's social competence. In other words, unlike previous studies suggesting that the high level of anger attribution bias and the low accuracy of emotional attribution are related to aggression [36], the results of this study can be examined in terms of several aspects as follows.

First, the negative correlation between children's emotional attribution accuracy and anger attribution bias was somewhat lower than in previous studies of US children [27]. This may be because emotional display rules in each culture are different. In individualistic cultures such as the United States, emotional expression is a voluntary expression of inner feelings, which is encouraged by parents [68, 69]. On the contrary, in a collectivist society, an individual's mood cannot be separated from a group's mood, and the control or suppression of emotions is valued and encouraged to maintain group harmony [70]. While American parents respond to their children's negative expressions and help them cope with their emotions, Asian parents typically want to predict and prevent children's negative emotional expressions [71]. In Asian cultures, which generally consider that anger promotes inner peace and social harmony, children tend to suppress their emotional expression [72, 73]. Schultz et al. argued that anger attribution bias plays an important role in social behavior by influencing the accuracy of attribution [37]. Accordingly, anger attribution bias has a relatively low correlation with the accuracy of emotional expression and does not exhibit a direct relationship with social competence.

Another possible reason for the discrepancy between the current and prior studies is that the sample size may have been too small and there was insufficient variance in anger attribution bias. Since this is the first study of Korean children, there is currently no evidence available by which to assess this further. Therefore, it is necessary to consider the relationship between anger attribution bias and social competence more carefully in future studies.

Schultz et al. argued that emotional attribution accuracy or anger attribution bias itself is not directly linked to prosocial behavior or aggression but rather is linked to social behavior in combination with other factors in the SIP process [36]. Although the integrated SIP model enhanced the understanding of children’s social competence by presenting an integrated perspective on the importance of emotion attribution, this tool was never empirically validated in the literature. Schultz et al.’s ACES [36] measures the encoding of cues, which is the first step of the integrated SIP model; thus, our Korean ACES can be understood as a useful tool for understanding Korean children’s social competence and SIP. Because we validated the structure of the original and our Korean ACES, we suggest that both emotion attribution and anger attribution be considered important parts of the first step of the integrated SIP model.

### Limitations

This study has several limitations. First, given that only six-year-olds were interviewed, we could only make limited assertions about children’s emotional development. Since emotion attribution accuracy begins to develop during early childhood and develops differently for diverse emotions, future studies should examine emotion attribution accuracy more extensively among different age groups and emotions, perhaps through a longitudinal study. Second, this study verified the validity and utility of a Korean ACES but failed to determine how children’s emotion attribution is related to actual social competence, as suggested by the integrated SIP model.

## Summary

It is not easy to assess how accurately young children recognize and understand other people’s emotions. Thus far, tools used to measure children’s emotional skills also measure their social and emotional abilities, such as social competence, rather than independently measuring emotional skills. The present study developed Korean ACES by modifying the original ACES which was introduced in the United States. The content validity of the revised Korean ACES was established via eight expert reviews. To test its reliability, the revised Korean ACES was conducted with 286 six-year-old children. Cronbach’s αs were 0.64 for the 40 items representing prototypical emotions and 0.64 for the 46 items measuring anger attribution. The results of the CFA show that the model fit was favorable—χ2/df = 1.547, RMSEA = 0.044 (0.000 to 0.089), TLI = 0.096, CFI = 0.985. We calculated descriptive statistics to examine the general tendencies of children’s emotional skills as specified by the Korean ACES. Participants could recognize emotions with about 76% accuracy from social situations, behaviors, and facial expressions showing prototypical emotions, and they could recognize anger in about 13% of facial expressions, behaviors, and situations that did not clearly express anger. The ACES score showed a weak positive correlation with self-recognition and expression ability (r = 0.20) and interpersonal ability to regulate others (r = 0.16) in emotional intelligence (for concurrent validity). The accuracy of emotion attribution was correlated with prosocial behavior (r = 0.13, p < 0.01), depression–isolation behavior (r = − 0.16, p < 0.05), and anger–aggression behavior (r = − 0.12, p < 0.05). The Korean ACES can stimulate further studies on Korean children’s emotional skills and contribute to various international collaborative studies that seek to compare the emotional skills of children from diverse cultural backgrounds. In follow-up studies, the ACES should be conducted with a wider age group, and the investigation should be expanded to each stage of SIP.
